# Atypical Presentation of Invasive Aspergillosis during Treatment with Mogamulizumab

**DOI:** 10.3390/jof10080584

**Published:** 2024-08-17

**Authors:** Paolo Pavone, Laura Arletti, Fiorella Ilariucci, Tommaso Albano, Deborah Lusetti, Romina Corsini, Francesco Merli, Sergio Mezzadri

**Affiliations:** Azienda USL—IRCCS di Reggio Emilia, 42123 Reggio Emilia, Emilia-Romagna, Italy; laura.arletti@ausl.re.it (L.A.); fiorella.ilariucci@ausl.re.it (F.I.); tommaso2407@gmail.com (T.A.); deborah.lusetti@gmail.com (D.L.); romina.corsini@ausl.re.it (R.C.); francesco.merli@ausl.re.it (F.M.); sergio.mezzadri@ausl.re.it (S.M.)

**Keywords:** aspergillosis, Mogamulizumab, lymphoma, CCR-4, immune reconstitution syndrome

## Abstract

Treatment with CCR-4 antagonists has been shown to be protective against the development of invasive pulmonary aspergillosis in animal models. Herein, we present a case of fatal invasive pulmonary aspergillosis in a patient receiving Mogamulizumab. A 64-year-old man with refractory mycosis fungoides was found to have diffuse bilateral pulmonary nodules during a chest CT in June 2022. Bronchoalveolar lavage (BAL) fungal and bacterial cultures and galactomannan were negative, as well as serum beta-glucan and galactomannan. Histology showed a lymphoid infiltrate with a negative fungal stain, so a presumptive diagnosis of lymphoma infiltration was made, and the patient started the CCR-4 antagonist Mogamulizumab treatment in August 2022. He had no symptoms until November when he presented to the hematology clinic reporting dyspnea. He had neutrophilic leukocytosis (18.610 cells/µL), his c-reactive protein was 27 mg/dL, and his skin lesions from mycosis fungoides were just starting to improve. A CT scan showed large diffuse bilateral severely necrotic cavitated lesions with thick walls and apparently synchronous evolution. Beta-glucan was 31 pg/mL (wako method), while serum galactomannan 3.6. BAL was positive for *Aspergillus fumigatus* culture and galactomannan. Patient started voriconazole but, despite being in a stable condition, he suddenly died after two days. Discussion: Paradoxically, worsening of the chronic pulmonary aspergillosis has been reported after nivolumab treatment, and immune reconstitution syndromes are usually seen during neutrophil recovery after intensive chemotherapy. Our patient already presented indolent lung lesions from 5 months before and he remained completely asymptomatic until the aspergillosis diagnosis when he quickly passed away. Even if a progression of the lesions was expected in 5 months, this case had an atypical presentation. During the 5-month period, he had no pulmonary symptoms, and his c-reactive protein was negative. Furthermore, in the setting of the natural progression of subacute/chronic aspergillosis, a different radiological picture was expected with a less severe and probably asynchronous evolution. We think that the immune restoration associated with Mogamulizumab (also supported by the concurrent clinical response of the skin lesions) could have been detrimental in this case, exacerbating a catastrophic immune response or alternatively masquerading the clinical progression of aspergillosis. Clinicians should be aware of immune reconstitution syndromes possibly leading to fatal outcomes in immunocompromised patients starting CCR-4 antagonists.

## 1. Introduction

In the setting of hematological malignancies, invasive pulmonary aspergillosis is usually associated with acute leukemia and bone marrow transplant. Patients affected by lymphoma are generally considered at low risk and even less involved when specifically looking at the patients with cutaneous T cell lymphomas [1].

We observed a disproportionately severe and fatal presentation of aspergillosis in a patient affected by mycosis fungoides, receiving treatment with the CCR-4 antagonist Mogamulizumab.

## 2. Case Presentation

**Figure 1:**
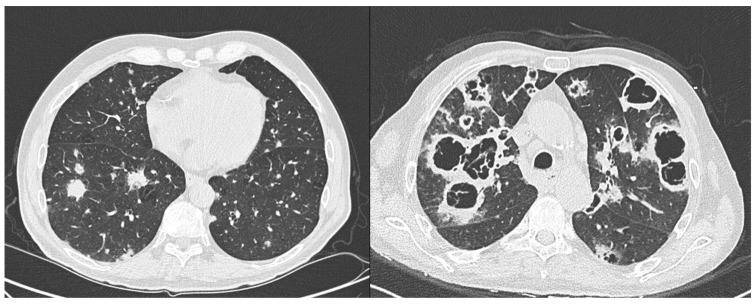
CT chest before and after Mogamulizumab treatment. Previously seen nodular lesions are substituted by massively necrotic and cavitated lesions.

In February 2019, a 65-year-old man was diagnosed with a folliculotropic variant of mycosis fungoides (MF). His primary comorbidity was peripheral vasculopathy, a condition linked to his history of heavy smoking. An unsuccessful therapeutic approach was first attempted with PUVA, followed by oral Bexarotene 150 mg per day, which was swiftly withheld because of hypertriglyceridemia. In July 2019, low-dose methotrexate (10 mg per week, then increased up to 30 mg per week) was started in association with radiotherapy for thoracic lesions. Following disease progression, the patient underwent Brentuximab Vedotin treatment (1.8 mg/mq every 21 days), completing 16 cycles in August 2021. Despite this treatment, only a partial response was achieved, due to the persistence of cutaneous lesions and PET-positive lymph nodes. Subsequently, between October 2021 and May 2022, the patient received liposomal doxorubicin (20 mg/mq on day 1 and 15), which did not yield significant benefit. A re-staging PET revealed persistent lymphonodal disease and the emergence of pulmonary widespread nodular lesions, which were later confirmed through a CT scan. To rule out infections, a bronchoalveolar lavage (BAL) was performed along with endoscopic biopsies. The BAL cytology was negative for both infectious agents and neoplastic cells. Additionally, fungal culture and galactomannan testing on the BAL sample turned out negative, as were the results of serum galactomannan and serum beta-d-glucan assays. The histological examination of a lung biopsy showed a lymphoid infiltrate with a negative fungal stain, leading to a presumptive diagnosis of lymphoma infiltration. Consequently, in August 2022, the patient started treatment with the CCR-4 antagonist Mogamulizumab (1 mg/kg on day 1, 8, 15, 22 on the first cycle then on day 1 and 15 of subsequent cycles of 28 days), no concomitant immune suppressive treatments were given. After 2 months of treatment, the skin lesions started to improve. In November 2022, the patient presented to the hematology clinic with a history of fever and dyspnea lasting for a few days. Notably, the c-reactive protein, which had previously been within normal range, was now elevated at 27 mg/dL. The patient also exhibited significant neutrophilia, indicating a heightened level of acute phase inflammation. A CT scan revealed the presence of large diffuse and severely necrotic cavitated lesions of the lungs, characterized by thick walls, nearly absent mycetomas, and synchronous evolution [Figure 1]. Serum galactomannan and beta-d-glucan tests yielded strongly positive results, where beta-d-glucan was 31 pg/mL (wako method) and serum galactomannan was 3.6. Furthermore, the BAL culture was positive for *A. fumigatus*, and the BAL galactomannan levels were also strongly elevated (>5 optical density index). Patient was started on voriconazole treatment p.o. and began oxygen supplementation 2 L/min. His conditions were stable but, after two days, he suddenly died. An autopsy was not performed.

## 3. Discussion

A paradoxical worsening of chronic pulmonary aspergillosis has been previously documented in patients undergoing Nivolumab treatment [2,3]. However, to the best of our knowledge, such a phenomenon has never been observed in patients receiving CCR-4 antagonist treatment. 

Our patient’s case presented indolent lung lesions that were misdiagnosed as lymphoma lung involvement. Subsequently, these lesions raised a strong suspicion of being *Aspergillus* nodules. Surprisingly, the patient remained completely asymptomatic for five months until the diagnosis of invasive aspergillosis, at which point his clinical condition deteriorated quickly, leading to his death. Our patient’s clinical evolution and severity of invasive aspergillosis were unexpected and appeared disproportionate according to the infection risk typically associated with the underlying hematologic disease. 

Patients with MF and Sezary syndrome (SS) are known to be at an increased risk of experiencing infectious complications. This heightened susceptibility often manifests as bacterial infections affecting soft tissue or skin, as well as instances of pneumonia [4]. Risk factors associated with infections are mainly represented by T cell depletion or dysfunction, as well as organ dysfunction [1]. Mogamulizumab is a recombinant humanized IgG1 monoclonal antibody designed to target CC chemokine receptor 4 (CCR4). It has received approval for previously treated MF and SS [5]. The principal adverse events related to Mogamulizumab treatment were infusion reactions and skin rashes, as reported in phase I/II [6] and MAVORIC trials [5]. Infectious complications were mainly represented by pneumonia and low-grade pyrexia according to the Common Terminology Criteria for Adverse Events (CTCAE). One case of *Pneumocystis jirovecii* pneumonia, categorized as a grade 3 CTCAE adverse event, was reported within the Mogamulizumab treatment arm. Also, real-world reports of Mogamulizumab utilization in the setting of cutaneous T-cell lymphoma did not indicate any case of invasive fungal infection [7,8]. 

In Japan, Mogamulizumab has obtained approval for the treatment of relapsed/refractory adult-onset acute lymphoblastic leukemia (AITL). Interestingly, in both the Phase II trial of Mogamulizumab used as a single agent for the treatment of AITL patients [9] and in a more recent randomized trial [10], no cases of invasive fungal infections were accounted for Mogamulizumab treatment. Finally, when Mogamulizumab was administered in combination with chemotherapy for the treatment of AITL in Japan, no documented case of invasive aspergillosis was described [11]. 

These observations indicate that Mogamulizumab, regardless of its use in various treatment contexts, did not appear to be linked to an increased risk of invasive fungal infections. Despite these evident findings derived from controlled clinical trials, the European Society of Clinical Microbiology and Infectious Diseases Consensus (ESCMID) has issued warnings regarding the potential of immune reconstitution syndromes in patients with infectious complications during Mogamulizumab treatment. These alerts are based on the mechanism of action of Mogamulizumab and its effects on the immune system [12]. In this context, the target of Mogamulizumab, CCR4, is not only highly expressed on neoplastic T lymphocytes of MF and SS but also on T-helper 2 (Th2) and regulatory T-cells (Tregs) [13], playing a pivotal physiological role. In vitro studies have demonstrated that CCR4 expression on Th2 lymphocytes is responsible for recruiting Th2 cells to the lung in allergic bronchopulmonary reactions in mouse models [14]. Furthermore, mice lacking CCR4 have shown a reduction in peribronchial and airway eosinophilia when stimulated with *A. fumigatus* conidia in models of allergic aspergillosis. These mice were also able to eliminate *A. fumigatus* conidia more rapidly than CCR4 naive mice [15]. This suggests that CCR4 absence could provide protective effects against allergic pulmonary reactions and fungal infection in mice. A recent study has further indicated that a CCR4-antagonist molecule known as SP50 is effective not only in preventing allergic aspergillosis but also in escaping invasive aspergillosis when inoculated with *A. fumigatus* lethal doses [16]. Taken together, these observations raise the chance that the CCR-4 may be involved in the control of aspergillus infection and that atypical clinical presentation of aspergillosis may occur if the CCR-4 pathway is altered. Noteworthy, patients receiving Mogamulizumab treatment exhibit a significant reduction in Tregs after just one course of treatment. This reduction occurs regardless of clinical response, as shown by Ni et al. [17]. 

The same study also revealed an increase in CD8+ T lymphocytes and natural killer (NK) cells, accompanied by an enhancement in the cytotoxic activity of NK cells. Indeed, on a positive note, another recent work has shown that long-term treatment with Mogamulizumab leads to the restoration of immunity in SS and MF patients through the elimination of exhausted T lymphocytes and the re-appearance of CD8 and CD4 memory T cells [18]. 

In the case of our patient, short-term Mogamulizumab therapy could have initially induced immune tolerance toward *A. fumigatus*, resulting in a decrease in the Th2 response. However, over time, this could have led to a dysregulated immune response to the pathogen due to the reduction in Tregs, the expansion of the CD8 and NK cells, and to the restoration of immunity. Unfortunately, given that we were not able to demonstrate evidence of a fungal infection in the pre-Mogamulizumab pulmonary nodules, we cannot truly disclose whether this was a newly acquired infection rather than IRIS.

Something that we also considered very atypical in the case of our patient, apart from the fast and fatal outcome, was the radiological presentation. Acute invasive pulmonary aspergillosis in patients with hematological malignancies is usually associated with the presence of ill-defined nodules with a halo sign during the neutropenic phase and subsequent cavitation during the neutrophil recovery due to the inflammation process and necrosis of the lesion. In this case, we observed diffuse and large cavitations with no signs of mycetoma inside the cavities and a very thick wall. In the setting of aspergillosis, the cavitation process is related to the presence of the inflammation and immune reaction of the host against the fungal mass thus leading to necrosis, but such a disproportionately cavitated and necrotic radiological presentation is not a common observation either after the neutrophil recovery during the induction of chemotherapy for acute myeloid leukemia.

We cannot disclose whether this infection was due to different *Aspergillus* species that are phenotypically indistinguishable by *A. fumigatus*. Morphological identification of *Aspergillus spp*. to the species level was accomplished using established procedures including microscopic and macroscopic characteristics. Colonies were typically blue-green with a suede-like surface, consisting of a dense felt of conidiophores. Conidial were columnar and uniseriate. We cannot exclude that *A. lentulus*, which is reported to be voriconazole-resistant could be the causative pathogen. Even if it is possible that we had misdiagnosed the species, the clinical presentation and evolution would not be justified only by the presence of *A. lentulus*. The patient died after only two days, so it was too early to correlate the outcome with the antifungal treatment. In summary, Mogamulizumab represents a valuable treatment opportunity for MF and SS patients due to its beneficial effect on disease control. However, the anti-CCR4 effects on Tregs, CD8+ T cells, and NK cells could potentially result in altered or uncontrolled immune response against infectious agents, similar to the immune reconstitution inflammatory syndrome described in HIV-positive patients and/or after neutrophil recovery [19,20]. Increased clinical vigilance for atypical presentation of opportunistic fungal infections is advised.

## 4. Conclusions

We observed an unexpected and quickly fatal pulmonary invasive aspergillosis during the course of Mogamulizumab treatment. Time from clinical presentation to death and radiological presentation were very atypical. Clinicians should be aware of possible atypical presentations of aspergillosis during treatment with CCR-4 antagonists.

## Data Availability

The original contributions presented in the study are included in the article, further inquiries can be directed to the corresponding author.
